# Metabolic Noise and Distinct Subpopulations Observed by Single Cell LAESI Mass Spectrometry of Plant Cells *in situ*

**DOI:** 10.3389/fpls.2018.01646

**Published:** 2018-11-15

**Authors:** Sylwia A. Stopka, Rikkita Khattar, Beverly J. Agtuca, Christopher R. Anderton, Ljiljana Paša-Tolić, Gary Stacey, Akos Vertes

**Affiliations:** ^1^Department of Chemistry, The George Washington University, Washington, DC, United States; ^2^Divisions of Plant Sciences and Biochemistry, C. S. Bond Life Sciences Center, University of Missouri, Columbia, MO, United States; ^3^Environmental Molecular Sciences Laboratory, Earth and Biological Sciences Directorate, Pacific Northwest National Laboratory, Richland, WA, United States

**Keywords:** single cell analysis, *in situ*, plant cells, heterogeneity, metabolic noise, mass spectrometry, metabolites, laser ablation electrospray ionization

## Abstract

Phenotypic variations and stochastic expression of transcripts, proteins, and metabolites in biological tissues lead to cellular heterogeneity. As a result, distinct cellular subpopulations emerge. They are characterized by different metabolite expression levels and by associated metabolic noise distributions. To capture these biological variations unperturbed, highly sensitive *in situ* analytical techniques are needed that can sample tissue embedded single cells with minimum sample preparation. Optical fiber-based laser ablation electrospray ionization mass spectrometry (f-LAESI-MS) is a promising tool for metabolic profiling of single cells under ambient conditions. Integration of this MS-based platform with fluorescence and brightfield microscopy provides the ability to target single cells of specific type and allows for the selection of rare cells, e.g., excretory idioblasts. Analysis of individual *Egeria densa* leaf blade cells (*n* = 103) by f-LAESI-MS revealed significant differences between the prespecified subpopulations of epidermal cells (*n* = 97) and excretory idioblasts (*n* = 6) that otherwise would have been masked by the population average. Primary metabolites, e.g., malate, aspartate, and ascorbate, as well as several glucosides were detected in higher abundance in the epidermal cells. The idioblasts contained lipids, e.g., PG(16:0/18:2), and triterpene saponins, e.g., medicoside I and azukisaponin I, and their isomers. Metabolic noise for the epidermal cells were compared to results for soybean (*Glycine max*) root nodule cells (*n* = 60) infected by rhizobia (*Bradyrhizobium japonicum*). Whereas some primary metabolites showed lower noise in the latter, both cell types exhibited higher noise for secondary metabolites. *Post hoc* grouping of epidermal and root nodule cells, based on the abundance distributions for certain metabolites (e.g., malate), enabled the discovery of cellular subpopulations characterized by different mean abundance values, and the magnitudes of the corresponding metabolic noise. Comparison of prespecified populations from epidermal cells of the closely related *E. densa* (*n* = 20) and *Elodea canadensis* (*n* = 20) revealed significant differences, e.g., higher sugar content in the former and higher levels of ascorbate in the latter, and the presence of species-specific metabolites. These results demonstrate that the f-LAESI-MS single cell analysis platform has the potential to explore cellular heterogeneity and metabolic noise for hundreds of tissue-embedded cells.

## Introduction

Cell types in tissues are distinguished by altered expression levels of transcripts, proteins, and metabolites resulting in their ability to serve specific functions. Dermal tissue in typical plant leaves consists of specific cell types, including epidermal cells (i.e., pavement and basal cells), trichomes, and guard cells. To facilitate their functions, different metabolic pathways are active in these cell types. Single cell analysis of some of these cell types revealed major differences in the abundances of primary and secondary metabolites. Compared to pavement and basal cells, the trichomes of *Arabidopsis thaliana* contained higher levels of metabolites from the kaempferol glycoside biosynthesis pathway (Zhang et al., 2014). Selectively analyzing specialized single plant cells, e.g., parenchyma cells, guard cells, trichomes, and excretory idioblasts (Foster, 1956; Valverde et al., 2001; Labhsetwar et al., 2014; Misra et al., 2015; Sibbitts et al., 2018) can provide insight into the biochemical processes and regulatory networks associated with their function.

Cellular heterogeneity within a particular cell type stems from stochastic expression of genes (Taniguchi et al., 2010; Brennecke et al., 2013; Kharchenko et al., 2014), the corresponding proteins (Newman et al., 2006), regulation of enzymes, asynchronous cell division, and epigenetic events. For example, in *Escherichia coli*, protein copy numbers follow gamma distributions and are uncorrelated with the corresponding mRNA copy numbers (Taniguchi et al., 2010). In *Saccharomyces cerevisiae*, the magnitude of proteomic noise is linked to the function of the protein, i.e., enzymes participating in protein synthesis exhibit low noise, whereas proteins that compensate for external perturbations show high noise (Newman et al., 2006).

Together with other contributing factors, these variations affect the concentration levels of downstream products, including metabolites (Altschuler and Wu, 2010; Paige et al., 2012; Bintu et al., 2016; Xiao et al., 2016; Rosenthal et al., 2018). These fluctuations propagate through the complex networks of regulatory cascades and they are collectively perceived as biological noise (Levine and Hwa, 2007; Labhsetwar et al., 2014; Forment and Rodrigo, 2017). Within a clonal population, two main sources can account for noise in gene expression; intrinsic noise, e.g., the stochasticity of transcription, mRNA production and destruction (Newman et al., 2006), and translation, and extrinsic noise that is caused by cell-to-cell-differences, e.g., the stage in cell cycle or the number of ribosomes in the cell (Elowitz et al., 2002; Swain et al., 2002).

Most biological experiments rely on bulk analysis that report on large cell population averages (millions of cells) and mask the presence of subpopulations, rare cells, and individual cellular variations. Measuring the distribution and the magnitude of biological noise is increasingly feasible through single cell analysis techniques, including fluorescence (Paige et al., 2012), microfluidics (Sibbitts et al., 2018), and Raman scattering (Kuku et al., 2017). The limited size and volume of single cells, and the low copy numbers of certain analytes make detection, identification, and quantitation challenging. To overcome these limitations, on the genomic and transcriptomic level nucleic acid amplification has been implemented (Efroni et al., 2015). However, this process cannot be applied in proteomics and metabolomics, as there is no amplification mechanism available for these biomolecules. The high sensitivity and selectivity of mass spectrometry (MS) make it the method of choice for metabolomic and proteomic analysis of single cells (Zenobi, 2013).

Due to the rapid turnover rates for some small molecules and the potential loss of material, sampling and handling of single cells requires special considerations. Several vacuum and ambient MS-based platforms have been developed to satisfy these requirements and explore cell-to-cell variations in tissue-embedded and circulating cells (Rubakhin et al., 2011; Yang et al., 2017; Zhang and Vertes, 2018). Matrix-assisted laser desorption ionization (MALDI) MS has been one of the predominant techniques for single cell analysis, utilizing different sampling methods, including laser capture micro dissection (Xu and Caprioli, 2002), isolation and extraction of single cells (Krismer et al., 2015), and microinjection of matrix directly into a cell (Agar et al., 2010). Time-of-flight secondary ion mass spectrometry (TOF-SIMS) has been used for chemical imaging of small biomolecules within cells and subcellular compartments (Barnes et al., 2012). However, vacuum methods require extensive sample preparation and dehydration, preventing analysis under native conditions.

Ambient single cell MS methods based on electrospray ionization (ESI) can sample cells within their natural environment and provide information on a less perturbed cell state. Probe-based ESI MS sampling techniques directly interrogate and analyze tissue-embedded cells by accessing intercellular material. These methods include probe sampling, e.g., live single cell MS (Fujii et al., 2015; Yang et al., 2017), capillary microsampling (Zhang et al., 2014), cell pressure probe MS (Nakashima et al., 2016), and single-probe MS (Gong et al., 2014). Another ambient single cell sampling method is based on laser ablation followed by ESI. These approaches include laser ablation electrospray ionization (LAESI) (Shrestha and Vertes, 2009), laser desorption ionization droplet delivery (LDIDD) (Lee et al., 2016), and laser ablation inductively coupled plasma (LA-ICP) ionization (Herrmann et al., 2017).

To achieve single cell resolution by LAESI, mid-IR laser pulses are coupled into an optical fiber with the distal tip etched to be commensurate with the size of a plant cell. Feasibility experiments were conducted on pigmented and non-pigmented *Allium cepa* epidermal cells (Shrestha and Vertes, 2009). Using this fiber-LAESI (f-LAESI), cell-by-cell molecular imaging of metabolites was demonstrated (Shrestha et al., 2011). Subcellular compartments of tissue embedded cells were also sampled and analyzed by f-LAESI by exposing the nucleus through microsurgery (Stolee et al., 2012).

In this contribution, we explore the abundance distributions of metabolites, their metabolic noise, detect the differences in prespecified and *post hoc* identified subpopulations, and demonstrate the analysis of rare cells by f-LAESI-MS. These single cell measurements are performed on a population of soybean (*Glycine max*) root nodule cells (*n* = 60) infected by rhizobia (*Bradyrhizobium japonicum*), and Brazilian waterweed (*Egeria densa*) cells (*n* = 103) consisting of mostly epidermal cells and some excretory idioblasts on the adaxial leaf surface. Epidermal cells from two closely related waterweed species, *E. densa* (*n* = 20) and *Elodea canadensis* (*n* = 20), were compared through single cell analysis by f-LAESI-MS to reveal significant differences between their metabolite compositions.

## Materials and Methods

### Fiber-LAESI

A Nd:YAG laser driven optical parametric oscillator (IR Opolette HE 2731; Opotek, Carlsbad, CA, United States) was used to generate mid-IR laser pulses of 2.94 μm wavelength, 7 ns pulse length, and 20 Hz repetition rate. The laser energy was externally attenuated to ∼1.3 ± 0.16 mJ, and this arrangement afforded a pulse-to-pulse stability of <5%. The laser light was focused through a 50-mm focal length plano-convex CaF_2_ lens directly onto the end of a 250-μm core diameter germanium oxide (GeO_2_) based optical fiber (HP Fiber, Infrared Fiber Systems, Inc., Silver Spring, MD, United States). For precise coupling, a fiber mount tilt stage (F-91TS, Newport, Irvine, CA, United States) was used that supported both the focusing lens and the bare fiber positioner (F-915T, Newport, Irvine, CA, United States).

The ends of a 1.0-m-long GeO_2_-based optical fiber were first stripped of the Hytrel and polyimide coatings by submersing both ends into 1-methyl-2-pyrrolidinone at 150°C for ∼2 min. Both ends were cleaved with a fiber cleaver for improved energy transmission. The end distal to the laser beam coupling was subjected to chemical etching by 4% HNO_3_ solution to form a tip commensurate in size with single cells. For uniform etching, a 100-mm diameter beaker was used that provided low curvature for the meniscus of the acid solution. The distal fiber end was lowered ∼0.5 mm deep into the solution. The etching was complete in ∼10 min when the liquid bridge between the solution surface and the fiber end was broken, resulting in a sharp tip. To remove chemical residues, both ends of the fiber were washed by deionized water prior to use. The end used for coupling the laser energy was secured into a bare fiber chuck (BFC300, Siskiyou Corporation, Grants Pass, OR, United States) and mounted in the fiber positioner. The etched end of the fiber was mounted onto a probe holder (MXP-150, Siskiyou Corporation, Grants Pass, OR, United States), attached to a micromanipulator (MN-151, Narishige, Tokyo, Japan) and steered into the field of view of the cell-targeting microscope (CTM) for sampling. Further details can be found in our previous work (Shrestha and Vertes, 2009).

### Microscope Visualization

Two separate visualization systems were used, one for monitoring the fiber tip to cell distance and the other for cell targeting. The CTM was positioned at a right angle from the sample surface and above the electrospray and MS inlet orifice axis. Brightfield illumination was provided by a white LED with 6500 K color temperature (MCWHL5, Thorlabs, Newton, NJ, United States) with its intensity adjusted by a LED driver (LEDD1B, Thorlabs, Newton, NJ, United States). For uniform lighting, an aspheric condenser lens with a diffuser surface (ACL2520-DG15-A, Thorlabs, Newton, NJ, United States) was installed. Since the illumination was perpendicular to the sample surface and the camera, a 30:70 (reflection:transmission) beamsplitter (BSS10R, Thorlabs, Newton, NJ, United States) was inserted into the optical train. For cell targeting, a 20× infinity-corrected objective lens (M Plan Apo, Mitutoyo Co., Kanagawa, Japan) was combined with a 1× tube lens and a 4-megapixel monochrome CCD camera (4070-GE, Thorlabs, Newton, NJ, United States). A long working distance fiber-monitoring microscope (FMM) (AM4815ZTL, Dino-lite, Torrance, CA, United States) with an extended magnification range (5× to 140×) was positioned at a 20° elevation angle to the sample surface.

### Plant Tissues

Live *E. densa* and *Elodea Canadensis* plants were obtained from the greenhouse of the Science and Engineering Hall at the George Washington University and from the Carolina Biological Supply Company (Burlington, NC, United States). Prior to analysis, healthy leaves were rinsed with HPLC grade deionized water. Using tweezers, a leaf was removed and placed onto a microscope slide abaxial side down.

Three-day-old soybean (*G. max*, Williams 82) seedlings were inoculated with *B. japonicum* (USDA110). After 21 days of growth, whole root nodules were harvested and flash frozen at -80°C. A more detailed description of soybean inoculation by rhizobia can be found in our previous work (Stopka et al., 2017). Intact frozen *G. max* root nodules were fixed in 2.5% carboxymethyl cellulose (CMC) embedding medium. Using a cryostat microtome (CM1800, Lecia Microsystems Inc., Nussloch, Germany) at -10°C, the frozen CMC block containing the nodule was sectioned at 30 μm thicknesses. The sections were thaw-mounted onto microscope slides and placed on the Peltier stage of the f-LAESI-MS system for analysis.

### Single Cell Sampling

To prevent the sample from drying, the microscope slide with the sample was mounted on a regulated Peltier stage that was set to 0 °C. A motorized XY-stage (MLS203, Thorlabs, Newton, NJ, United States) and a piezoelectric actuator (PIAK10, Thorlabs, Newton, NJ, United States) in the *Z* direction were used to adjust the position of the cooling stage. With the fiber tip in the field of view of the CTM, XY adjustments were made to the sample position, so that the fiber tip was directly over a cell selected for analysis. The sampling area on the leaf was in the basal region, at 4–5 mm from the stem. Then, the sample stage was driven in the *Z* direction until the fiber was just touching the cell surface. The laser was then fired for ∼1 s (∼20 pulses) that ejected the contents of the cell as a plume of fine particulates. The ejection plume was intercepted by an electrospray that was on axis with the inlet orifice of the mass spectrometer (Synapt G2-S, Waters, Milford, MA, United States). The analysis was performed in negative ion mode with a spray solution composition of 2:1 (v/v) MeOH:CHCl_3_, a flow rate of 500 nL/min, and a spray voltage of 2.7 kV.

### Statistical and Data Analysis

A total of 103 (*n* = 97 epidermal cells and *n* = 6 idioblasts) single cell spectra were collected from 16 leaves of the *E. densa* species under identical instrumental settings and environmental conditions. The raw files have been uploaded with Study Identifier MTBLS765 to MetaboLights,^1^ a web-based metabolomics repository. All raw data files were processed by removing the ESI background ions through spectral subtraction to reveal the sample related peaks (MassLynx, 4.1, Waters, Milford, MA, United States). Data reduction was achieved by peak picking and deisotoping using mMass (Strohalm et al., 2010). The web-based metabolomic processing software, MetaboAnalyst 3.0 was utilized for multivariate statistical analysis to explore differences between the epidermal vs. idioblast cell types (*n* = 6 each) and the *E. densa vs. E. canadensis* epidermal cells (*n* = 20 each). For this analysis, the data were normalized by the sum of the cell-related spectral intensities and Pareto scaling was performed. Cutoff values for the volcano plots were set to 1.0 and -1.0 on log_2_ scale, corresponding to 2.0 and, 0.5 for the fold change values, and 0.05 for the *p*-value for statistical significance. Box and whisker plots were constructed by plotting the relative intensities within each sample group. Peak assignments were based on accurate mass measurements and comparison with online databases (Plant Metabolic Network,^2^ last accessed 7/6/2018, and METLIN,^3^ last accessed 7/6/2018) (Guijas et al., 2018). To support metabolite identification, tandem MS was performed using data dependent acquisition on leaf blade and root nodule extracts through direct infusion. Collision-induced dissociation (CID) was used with energies ramped from 10 to 55 eV. The corresponding fragments were compared to the external databases above and our internal LAESI metabolite database.

For low abundance species, the signal occasionally dropped below the limit of detection, and no peak was detected. In the classification of missing values, this corresponds to a left-censored missing not at random (MNAR) case. For such datasets, imputation of the missing values can be best handled by quantile regression imputation of left-censored data (QRILC) (Wei et al., 2018). The web-based missing value imputation tool for MS-based metabolomics data was used for ions with at least 80% of non-zero data present^4^ (last accessed 7/24/2018).

## Results

### Single Cell Sampling

**FIGURE 1:**
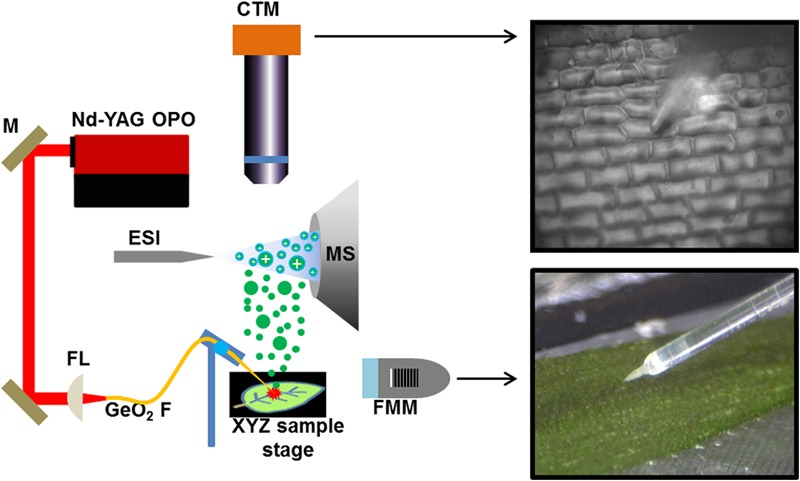
Schematic of f-LAESI setup for single cell analysis. A mid IR laser beam is steered by gold coated mirrors (M) and coupled through a CaF_2_ focusing lens (FL) into a germanium oxide-based optical fiber (GeO_2_ F). When the etched fiber tip is brought into close proximity of the cell, an ablation plume of neutrals (green dots) is produced. The expanding plume is intercepted and ionized by an electrospray (blue dots) that is on axis with the inlet orifice of the mass spectrometer. Both the optical fiber and the sample are mounted on XYZ stages for fine adjustments. A fiber monitoring microscope (FMM) is positioned under 20° elevation angle to monitor the distance between the fiber tip and the sample surface. For cell selection, a cell targeting microscope (CTM) is positioned under right angle to the sample surface.

Previously, we demonstrated that single plant cell analysis can be achieved by f-LAESI-MS with ∼30 μm spatial resolution (Shrestha and Vertes, 2009). Using a reduced diameter (250-μm core) GeO_2_-based optical fiber with a tip radius of curvature of *R* = ∼5 μm, improved the spatial resolution to ∼15 μm. This allowed for precise sampling of the cells without partially ablating the adjacent cells. The adaxial epidermal cell dimensions for *E. densa* were 140 μm × 40 μm × 40 μm, in length, width, and depth, respectively, corresponding to a volume of ∼220 pL, whereas for *G. max*, the cell dimensions of 50 μm × 50 μm × 30 μm in the tissue section defined a volume of ∼75 pL. The etched optical fiber tip was brought into gentle contact with the surface of the cell avoiding its mechanical disruption. When the laser was fired, the cell wall and plasma membrane were ruptured, and the cell content was ejected into the electrospray of the f-LAESI source. The schematic of single cell analysis by f-LAESI-MS is shown in Figure 1.

**FIGURE 2:**
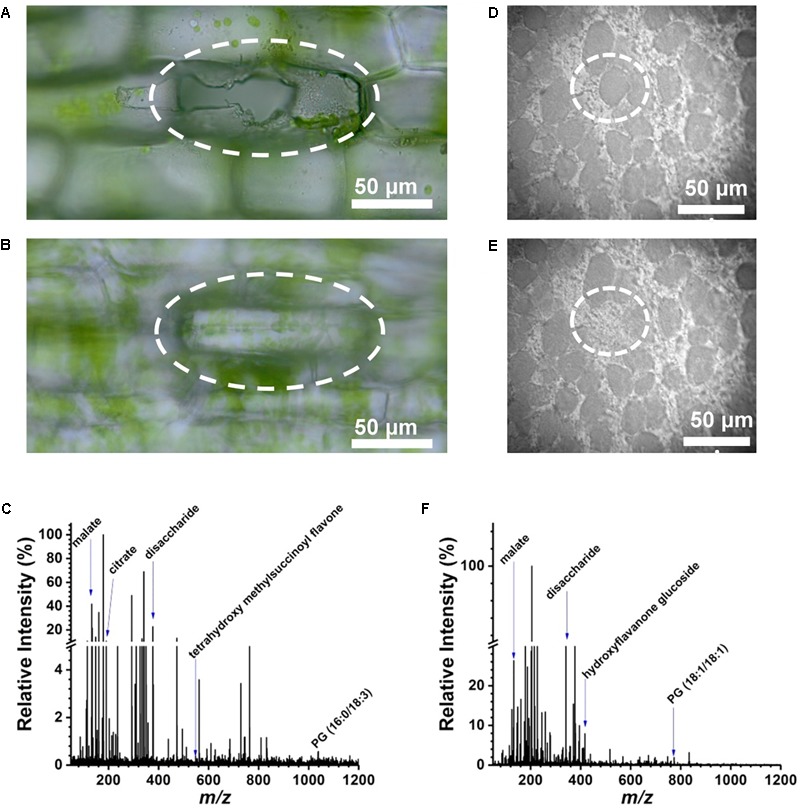
Selective cell sampling in leaf tissue composed of two cell layers. Focusing on **(A)** adaxial cell layer and **(B)** abaxial cell layer of an *E. densa* leaf blade after an ablation event reveals that only the adaxial cell is sampled. **(C)** Corresponding negative ion mode mass spectrum from a single cell that exhibits ∼186 spectral features with detected biomolecules ranging from amino acids to secondary metabolites. Microscope image of single infected cell in *G. max* root nodule section **(D)** before and **(E)** after ablation of circled cell. **(F)** Negative ion mass spectrum of the ablated *G. max* cell.

An *E. densa* leaf blade consists of two epidermal cell layers without the presence of mesophyll in this tissue. Away from the midvein, epidermal cells (*n* = 97) and excretory idioblasts (*n* = 6) were sampled from the adaxial side. The CTM was used to select the cells for analysis (Figure 2A) and to image the interrogated area after the ablation to confirm that only individual cells were ablated (see Figure 2B). Offline optical imaging of both epidermal layers was performed to assess the damage to the underlying and neighboring cells after ablation. In the adaxial layer, only the targeted cell was ruptured (see Figure 2A), whereas in the underlying abaxial layer, all the cells were intact (see Figure 2B). Large infected cells in the *G. max* root nodule sections were interspersed with smaller uninfected cells (see Figure 2D). The two cell types were clearly distinguished in the microscope image based on the size difference. Using the CTM only infected cells were selected for fiber ablation, before (Figure 2D) and after (Figure 2E) microscope images of the ablation event revealed only the cell of interest to be sampled.

**FIGURE 3:**
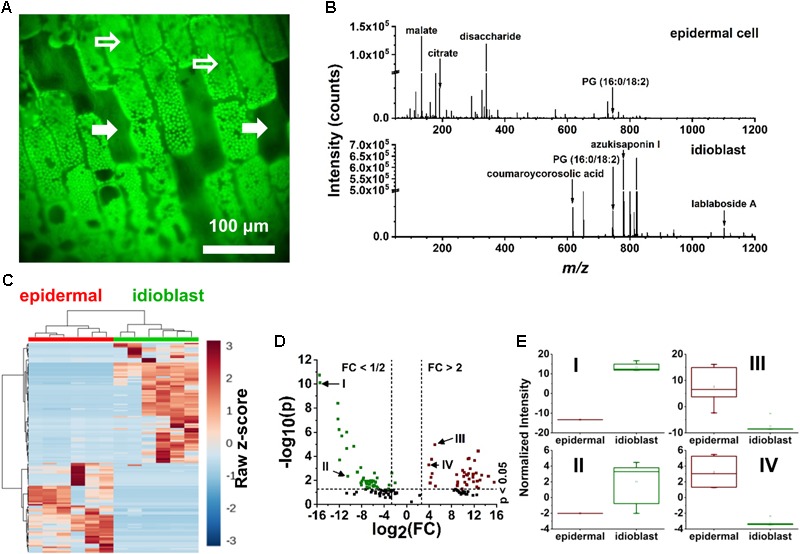
Localization of idioblast and spectral variance within the *E. densa* single epidermal and idioblast cells from a whole leaf tissue. **(A)** An optical image from the CTM microscope with the blue illumination conditions. Epidermal cells (indicated by open arrows) contain chloroplasts, whereas idioblasts lack chloroplast and are non-fluorescent (close arrows). **(B)** A typical negative ion mode mass spectrum from (top) an epidermal cell and (bottom) idioblast cell, indicating the metabolite differences. **(C)** A heat map consisting of (*n* = 6 each) epidermal and idioblast single cells to illustrate the clustering of spectral features from the mass spectra, where each column is a spectrum and each row is an *m/z* value. **(D)** Volcano plot of significantly different spectral features from (green) idioblasts and (red) epidermal cells. For example, (I) azukisaponin I and (II) coumaroylcorosolic acid were found to be more abundant in idioblasts, whereas (III) citrate and (IV) malate are at higher level in epidermal cells. The threshold values of a fold change >2 and the *p*-value <0.05 based on the Student’s *t*-test were only considered. **(E)** Four significant species that were identified from the volcano plot, (I) azukisaponin I, (II) coumaroylcorosolic acid, (III) citrate, and (IV) malate.

Specialized excretory idioblasts, sparsely interspersed throughout the *E. densa* leaf blade, were present in much lower numbers than epidermal cells, and did not contain chloroplasts. To pinpoint the idioblasts for analysis, fluorescence imaging tailored to chloroplast autofluorescence was used to locate these non-fluorescent cells. A blue shifted LED light source with an illumination maximum at 470 nm and an emission band at 620–700 nm were selected to highlight the chloroplast containing epidermal cells. The embedded excretory idioblasts were targeted for ablation based on the lack of fluorescent chloroplasts in these cells (see the solid arrows in Figure 3A).

### Single Plant Cell Mass Spectrometry

To make sure the efficiency of ablation had little to no effect on the spectra, the fiber tip was positioned over the centroid of the targeted cell using CTM visualization. This way, the effect of the cell walls on the ablation was minimized. The volumes of excretory idioblasts and epidermal cells were similar. Visualization by the CTM confirmed that the cell contents from both cell types were fully ablated.

From the *E. densa* epidermal cells, on average ∼186 spectral features were observed after deisotoping (Figure 2C and Figure 3B, top panel). For example, of the 20 common amino acids, the following six were detected: [alanine-H]^-^ at *m/z* 88.035, [leucine/isoleucine-H]^-^ at *m/z* 130.084, [asparagine-H]^-^ at *m/z* 131.037, [aspartate-H]^-^ at *m/z* 132.024, [histidine-H]^-^ at *m/z* 154.062, and [tryptophan-H]^-^ at *m/z* 203.084. Other primary metabolites that were detected from the epidermal cells included [pyruvate-H]^-^ at *m/z* 87.005, [malate-H]^-^ at *m/z* 133.013, [citrate-H]^-^ at *m/z* 191.012, [glutamate-H]^-^ at *m/z* 146.044, and [disaccharide-H]^-^ at *m/z* 341.109. Secondary metabolites, including flavonoids and flavonoid glucosides, e.g., [dihydroxy methylflavan-H]^-^ at *m/z* 255.095, [luteolin/kaempferol-H]^-^ at *m/z* 285.044, [tetrahydroxy methylsuccinoyl flavone-H]^-^ at *m/z* 399.072, and [luteolin/kaempferol glucuronide-H]^-^ at *m/z* 461.082 were also identified. Within the epidermal cells some glycerophospholipids, e.g., phosphatidylglycerol (PG), [PG (16:0/18:2)-H]^-^ at *m/z* 745.493, and phosphatidylinositol (PI), [PI (16:0/18:2)-H]^-^ at *m/z* 831.504, were detected.

The mass spectra of excretory idioblast contained ∼68 spectral features mostly populating the intermediate *m/z* 600–1200 range (bottom panel of Figure 3B). The metabolic makeup of idioblasts, detected by single cell f-LAESI-MS, contained lipids, triterpenoids, and triterpene saponins. In the lower *m/z* range, some metabolites detected in the epidermal cells were also present in the idioblasts, but with significantly lower abundance (see Figures 3C–E). For example, malate exhibited an *I*_idioblast_/*I*_epidermal_ ratio of 0.03, indicating a more biologically driven need of malate in the epidermal cells. A few other primary metabolites with low abundance were observed in the idioblasts including asparagine (*I*_idioblast_/*I*_epidermal_ = 0.02), and citrate (*I*_idioblast_/*I*_epidermal_ = 0.04). The PG(16:0/18:2) lipid species (*I*_idioblast_/*I*_epidermal_ = 1.77) and medicoside I triterpene saponin (*I*_idioblast_/*I*_epidermal_ = 70.48) were observed in both cell types, however, with much higher abundance in the idioblasts. Unique predominantly secondary idioblast metabolites, for example, [coumaroylcorosolic acid-H]^-^ at *m/z* 617.394, [azukisaponin I-H]^-^ at *m/z* 799.452, and [lablaboside A-H]^-^ at *m/z* 1101.555, were also observed.

After deisotoping, a representative *G. max* single cell mass spectrum consisted of ∼157 spectral features (see Figure 2F). Several different classes of compounds were present, ranging from amino acids, to other primary metabolites, flavonoids, flavonoid glucosides, and lipid species. Ions of some small molecules, e.g., [oxalate-H]^-^ at *m/z* 89.990, [fumarate-H]^-^ at *m/z* 115.003, [malate-H]^-^ at *m/z* 133.0142, [ascorbate-H]^-^ at *m/z* 175.029, and [citrate-H]^-^ at *m/z* 191.016, were observed both in the *G. max* and the *E. densa* cells. A few secondary metabolites were annotated in the *G. max* cells included [hydroxyflavanone glucoside-H]^-^ at *m/z* 417.090, [trihydroxyflavone glucoside-H]^-^ at *m/z* 431.136, and [PG (18:1/18:1)-H]^-^ at *m/z* 773.539. All of the metabolite assignments mentioned here aligned well with our previous conventional LAESI-MS analysis of whole soybean root nodules, and MALDI-MS imaging of nodule sections (Stopka et al., 2017; Veličković et al., 2018).

### Technical vs. Metabolic Noise

Technical noise is the result of signal fluctuation attributable to the analytical technique. This technical noise is connected to signal intensity variations. It is different from the more commonly discussed background noise that stems from electronic and chemical interferences appearing as a fluctuation in the baseline. The measured signal always satisfied the **S*/*N* >3* requirement. To distinguish technical variability from the metabolic noise, i.e., signal variation of biological origin, a homogeneous solution standard of an endogenous compound was sampled using the same experimental conditions as in single cell analysis. Signal intensities from *n* = 15 replicates were determined by f-LAESI-MS by ablating 1 μL droplets of a 500-μM glutamate standard solution. To prevent material loss and minimize droplet fission, the 1-μL aliquots were deposited onto Parafilm M and directly ablated by the optical fiber tip. In-source fragmentation at 23 ± 4% level was observed in the spectra of the glutamate standard. To assess technical variability, the intensities of the precursor ion at *m/z* 146.041 and the two in-source product ions at *m/z* 128.040 and 102.059 were used to calculate total glutamate intensities. The normalized intensities from droplet standards were characterized by a mean of μ*_t_* = 16.6, a standard deviation of σ*_t_* = 2.5, and a coefficient of variation (COV = 100^∗^σ_t_/μ_t_) of 14.9% corresponding to a technical noise of $\eta_{t}^{2}$ = $\sigma_{t}^{2}$/$\mu_{t}^{2}$ = 0.02 (Supplementary Figure S1). The data were consistent with a normal distribution with a coefficient of determination *R*^2^ = 0.92.

**FIGURE 4:**
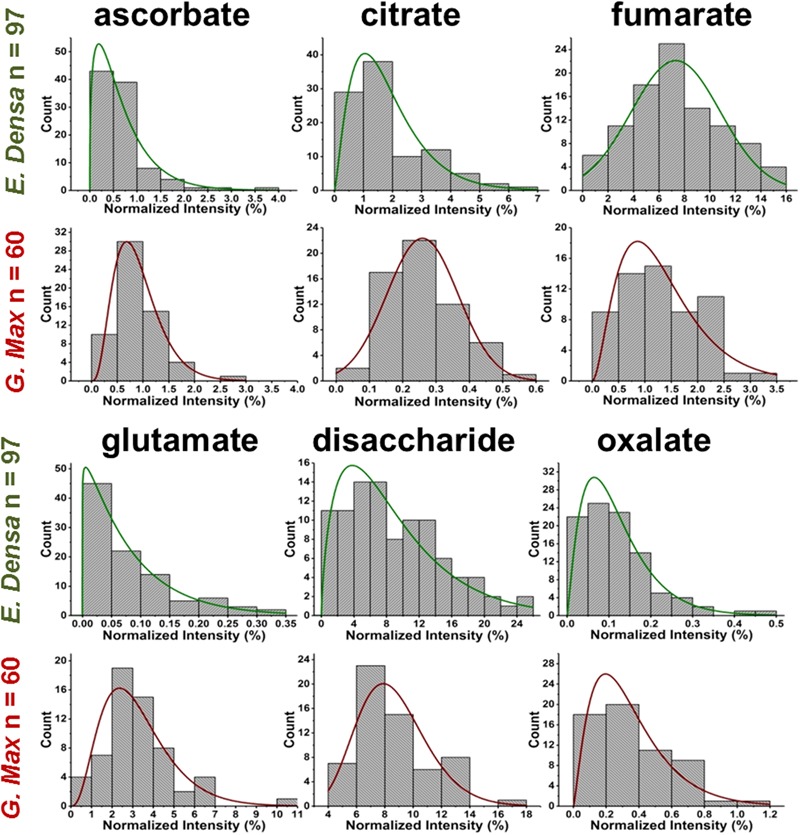
Metabolite abundance distributions for *E. densa* epidermal cells (first and third rows), and *G. max* infected root nodule cells (second and fourth rows). Data are consistent with gamma and normal distributions.

In relative terms, the signal variability of glutamate from single *E. densa* epidermal cells (*n* = 97) and *G. max* infected root nodule cells (*n* = 60) expressed in normalized intensities yielded μ_m_ = 0.083, a σ_m_ = 0.080, and a COV of 97.3% with a measured noise of $\eta_{m}^{2}$ = $\sigma_{m}^{2}$/$\mu_{m}^{2}$ = 0.94, and μ_m_ = 3.23, σ_m_ = 1.63, and a COV of 50.7% with a measured noise of $\eta_{m}^{2}$ = $\sigma_{m}^{2}$/$\mu_{m}^{2}$ = 0.27, respectively (see Figure 4). The measured noise originates from a combination of metabolic and technical noise. The technical noise, found to be $\eta_{t}^{2}$ = $\sigma_{t}^{2}$/$\mu_{t}^{2}$ = 0.02, did not significantly affect the determination of metabolic noise and cellular heterogeneity for either of the studied systems.

The glutamate intensity distributions were consistent with gamma distributions characterized by a probability density function of:

(1) $$f(x)=\frac{\beta^{\alpha }}{\Gamma (\alpha )}x^{\alpha -1}e^{-\beta x},$$

where α and β are the shape and rate parameters, respectively, and Γ(α) is the gamma function. They are directly related to the inverse of metabolic noise, α = μ^2^/σ^2^, and to the Fano factor, β = σ^2^/μ. Although a lognormal distribution could also be used to describe these data, the gamma distribution was consistent with more of the metabolite data, and it was also used in the literature to describe protein copy numbers (Taniguchi et al., 2010).

### Prespecified Subpopulations

**FIGURE 5:**
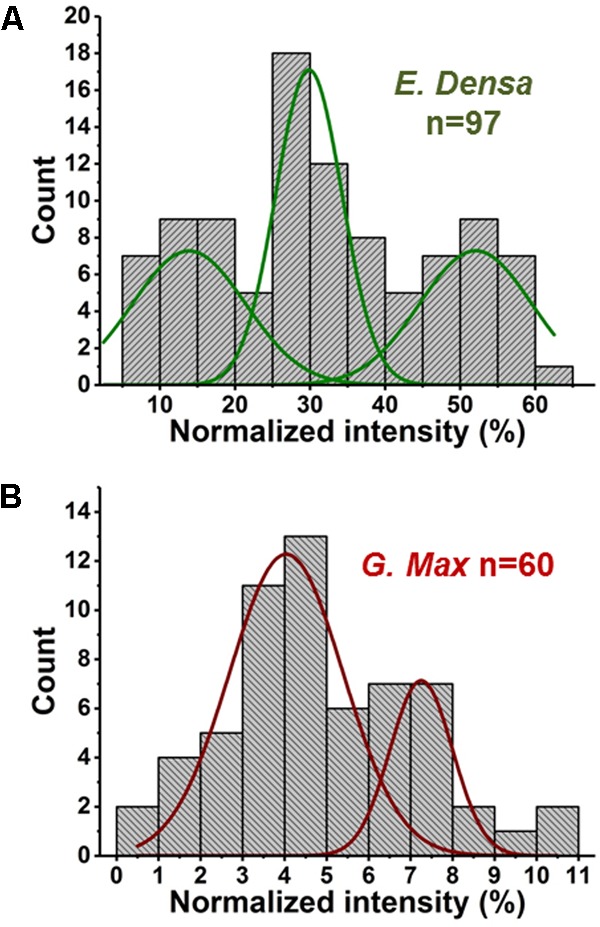
Abundances of malate in **(A)** *E. densa* epidermal cells and **(B)** *G. max*-infected root nodule cells exhibit trimodal and bimodal distributions, respectively. Deconvolution using normal distributions yielded the corresponding mean values and standard deviations: (*top panel*) μ_1_ = 14.0 and σ_1_ = 6.4, μ_2_ = 29.8 and σ_2_ = 3.8, and μ_3_ = 52.0 and σ_3_ = 6.3, and (*bottom panel*) μ_1_ = 4.0 and σ_1_ = 1.1 and μ_2_ = 7.5 and σ_2_ = 0.6.

Variations of primary and secondary metabolite levels and lipids were observed for a total of *n* = 103 cells (*n* = 97 epidermal cells and *n* = 6 idioblasts) in *E. densa* leaves (see six examples in Figure 4). As these two cell types were prespecified based on their phenotypic differences, we collected and processed the data separately for them. Metabolite ion intensities were normalized by the sum of all cell-related ion intensities in the single cell spectra. For the metabolites with significant abundance values extending down to zero, such as ascorbate, glutamate, and disaccharide, the counts followed a gamma distribution. For metabolites that were present at significant levels in all cells, e.g., fumarate (see Figure 4) and malate (see Figure 5), the counts followed normal distributions or multimodal distributions. In malate, the latter were deconvoluted into a combination of three normal distributions.

For some metabolite levels, there were significant differences between the epidermal cells and the idioblasts. For example, based on *t*-tests disaccharide (*p* = 0.001) and citrate (*p* = 0.001) was significantly higher in the epidermal cells. For malate, medicoside I, and azukisaponin I, the metabolite levels in idioblasts were dramatically different from epidermal cells. Malate was almost absent in idioblasts putting their levels outside the range found in epidermal cells. Conversely, medicoside I and azukisaponin I were produced at very low levels by epidermal cells and at significantly higher levels by idioblasts. The very low level, or absence, of several primary metabolites in idioblasts might be correlated with their biological function. As excretory cells, they perform reduced levels of biosynthesis, and mostly retain the functions needed for secretion and storage of certain compounds.

For *G. max*, there was only one prespecified population of cells, the infected root nodule cells, and normal distributions were found to fit the data for all the related metabolites in Figure 4. Supplementary Tables S1–S3 present the descriptive statistics, the goodness of fit for normal, lognormal and gamma distributions, and the regression parameters for all successful fits to the *E. densa* and *G. max* data, respectively.

### *Post hoc* Subpopulations

In two cases, malate distributions for *E. densa* and *G. max*, trimodal and bimodal distributions were found, respectively. They were deconvoluted into a combination of normal distributions (see Figure 5). Such deconvolution enabled the *post hoc* grouping of epidermal cells into subpopulations with low, intermediate, and high levels of malate. The mean values and standard deviations for the normally distributed subpopulations in *E. densa* were μ_1_ = 14.0 and σ_1_ = 6.4, μ_2_ = 29.8 and σ_2_ = 3.8, and μ_3_ = 52.0 and σ_3_ = 6.3, respectively. For *G. max*, the two subpopulations were characterized by μ_1_ = 4.0 and σ_1_ = 1.1 and μ_2_ = 7.5 and σ_2_ = 0.6. To confirm the presence of subpopulations, the *D* > 2 Ashman’s criterion for normally distributed components was used, where

(2) $$D_{12}=\frac{\sqrt{2}|\mu_{1}-\mu_{2}|}{\sqrt{\sigma_{1}^{2}+\sigma_{2}^{2}}}$$

(Ashman et al., 1994). According to this criterion, the three components in the malate heterogeneity in *E. densa* are characterized by *D*_12_ = 3.04 and *D*_23_ = 4.26, whereas in *G. max*, the two components satisfy *D*_12_ = 3.46. This means that, based on their malate levels, the *E. densa* epidermal cells and the *G. max* infected root nodule cells can be *post hoc* grouped into three and two subpopulations, respectively. If the multimodal malate distribution was a consequence of cell morphology differences, other metabolites would also exhibit such distributions. Yet none of the other studied metabolites showed multimodality. Thus, it is unlikely that the bi-/tri-normal malate distributions are the result of differences in cell morphology or ablation efficiency.

### Metabolic Noise

Metabolic noise is induced by variations in enzyme levels, metabolic fluxes, metabolite pool sizes, and environmental factors. For most metabolites, these quantities are not available. Nevertheless, from single cell measurements we can estimate the amplitude of metabolic noise. Table 1 compares the metabolic noise for primary and secondary metabolites and lipids, for *E. densa* leaf epidermal cells and *G. max*-infected root nodule cells. In general, primary metabolites exhibit lower metabolic noise compared to lipids and secondary metabolites. Comparing noise for primary metabolites from the two plants revealed higher amplitudes in *E. densa.* The noise of lipid levels seems to be consistent between the two plant types. Looking at the correlation between the types of distributions and the amplitude of the noise, we found that metabolite levels that follow normal distributions are associated with lower metabolic noise.

**Table 1:** Measured metabolic noise, $\eta_{m}^{2}$ = $\sigma_{m}^{2}$/$\mu_{m}^{2}$, in *E. densa* leaf epidermal cells (*n* = 97) and *G. max*-infected root nodule cells (*n* = 60).

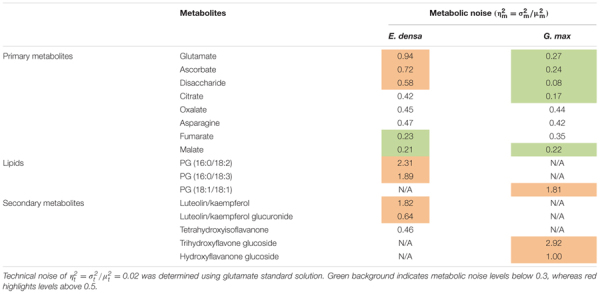

### Comparison of *E. densa* and *Elodea canadensis* Epidermal Cells

**FIGURE 6:**
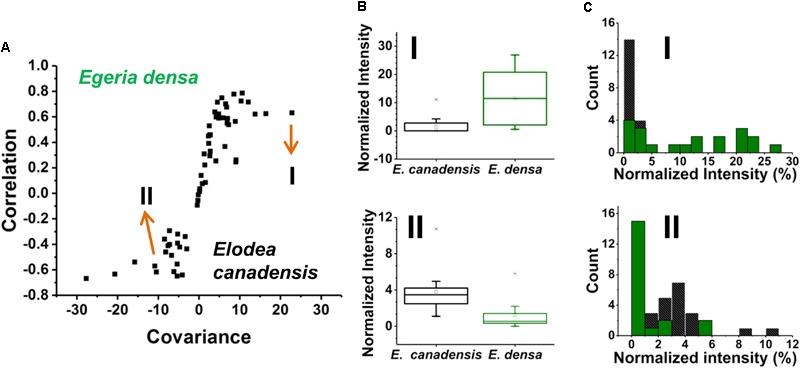
**(A)** An S-plot showing the variance between the two populations of single epidermal cells (*n* = 20 each) in which the metabolites on the wings of the S-plot have the highest covariance and correlation. **(B)** Box and whisker plots of two metabolites that showed high covariance and correlation values from the S-plot. (*top panel*) a hexose found higher in abundance in *E. densa* with a *p*-value of 0.0001 and (*bottom panel*) ascorbate intensity was higher in the *E. canadensis* epidermal cells with a *p*-value 0.0002. Both *p*-values were based on the Student’s *t*-test. **(C)** Metabolite abundance distributions for both (*top panel*) hexose and (*bottom panel*) ascorbate are displayed in (black) *E. canadensis* and (green) *E. densa*.

Prespecified populations of epidermal cells from the leaves of two closely related waterweed species, *E. densa* and *E. canadensis* were analyzed and their metabolite compositions were compared. The epidermal cell volumes of the *E. densa* and *E. canadensis* were comparable at 245 ± 68 and 229 ± 71 pL, respectively. In both cases, the analysis consisted of sampling most of the cell content under standardized conditions. A total of *n* = 20 individual cells was analyzed from each species, and orthogonal projections of latent structures discriminant analysis (OPLS-DA) was performed. The resulting S-plot is shown in Figure 6A. The peaks responsible for most of the variance between the spectra from the two species with high covariance and correlation were found on the wings of the S-plot.

A few unique spectral features were only observed in the *E. canadensis* epidermal cells, e.g., *m/z* 637.105 (luteolin/kaempferol digalacturonide), *m/z* 318.035, and *m/z* 677.267, the latter two with unknown annotation. Metabolites with higher abundances detected in *E. densa* include malate (I*_E._*_densa_/I*_E. canadensis_* = 1.30), glutamate (I*_E._*_densa_/I*_E. canadensis_* = 1.15), hexose (I*_E. densa_*/I*_E. canadensis_* = 8.15), and threonate (I*_E._*_densa_/I*_E. canadensis_* = 30.66) (see Figure 6B top panel). In the *E. canadensis* cells, ascorbate (I*_E._*_densa_/I*_E. canadensis_* = 0.31) and luteolin/kaempferol (I*_E. densa_*/I*_E. canadensis_* = 0.008) were observed at higher signal intensities (see Figure 6B lower panel).

The metabolic noise of some biomolecules detected in the two plants was compared. For example, hexose was found more abundant in *E. densa* and this was reflected in the metabolite level distributions for the two plants (see the top panel of Figure 6C). In the *E. canadensis* cells, the lower mean of hexose intensities, μ_m1_ = 1.75, were accompanied by a relatively large standard deviation, σ_m1_ = 2.56, resulting in a metabolic noise of $\eta_{\mathrm{m1}}^{2}$ = $\sigma_{\mathrm{m1}}^{2}$/$\mu_{\mathrm{m1}}^{2}$ = 2.14. For *E. densa*, the higher mean value for the hexose level, μ_m2_ = 11.57, and relatively lower standard deviation, σ_m2_ = 9.22, yields a significantly lower metabolic nose, $\eta_{\mathrm{m2}}^{2}$ = $\sigma_{\mathrm{m2}}^{2}$/$\mu_{\mathrm{m2}}^{2}$ = 0.64. Conversely, ascorbate was detected at higher intensities in the *E. canadensis* cells. The higher mean of ascorbate intensities, μ_m1_ = 3.81, were accompanied by a relatively large standard deviation, σ_m1_ = 2.29, resulting in a low metabolic noise of $\eta_{\mathrm{m1}}^{2}$ = $\sigma_{\mathrm{m1}}^{2}$/$\mu_{\mathrm{m1}}^{2}$ = 0.36. For *E. densa*, the lower mean value for the ascorbate level, μ_m2_ = 1.23, and relatively lower standard deviation, σ_m2_ = 1.64, yields a significantly higher metabolic noise of $\eta_{\mathrm{m2}}^{2}$ = $\sigma_{\mathrm{m2}}^{2}$/$\mu_{\mathrm{m2}}^{2}$ = 1.76.

## Discussion

Integrating fluorescence and brightfield microscopy with f-LAESI-MS for single cell analysis allows the selective targeting of specialized or rare cells. This multimodal system offers unique capabilities to target cells labeled by a fluorescent tag or identified by cell morphology for single cell analysis. Although the system has been used to analyze relatively large plant cells with volumes of 75–250 pL, typical animal cells with volumes of low picoliters are much smaller so their analysis remains a challenge. Possible mitigation strategies include increasing the sampling, ionization, and ion collection efficiencies for the LAESI-MS analysis.

Here, we demonstrated the direct analysis of idioblasts interspersed among epidermal cells in *E. densa* leaf tissues. Idioblasts can be identified based on their autofluorescence in the UV range (Hara et al., 2015). The visualization of these cells for our study was based on imaging the autofluorescence of chlorophyll *a* (Krause and Weis, 1991). Since the idioblasts do not contain chloroplasts, they were identified and targeted based on the lack of fluorescence (Figure 3A).

Excretory idioblasts in plant tissues have generated interest due to the natural products stored within these specialized cells with potential industrial and medicinal value. However, these cells occur in significantly lower numbers than epidermal cells in plant tissues. Assuming a leaf surface area of ∼43.5 ± 15 mm^2^, the estimated numbers of adaxial epidermal cells and idioblasts are ∼7700 and 214, respectively (Hara et al., 2015). This means a 2.8% frequency for the idioblasts resulting in a 36-time dilution of the analytical signal for them in a bulk sample. Through plasmolysis, it was demonstrated that only half the cell volume of an *E. densa* leaf idioblast contains water as compared to nearly the whole cell volume in epidermal cells (Cordes William, 2006). The detailed composition of *E. densa* idioblasts is still unknown but it is reported to contain lipids, tannins, oils, and enzymes such as lipase (Cordes William, 2006). In our study, lipids and triterpene saponins were detected along with several unknown compounds that were only characterized by their accurate mass. For example, singly charged ions at monoisotopic *m/z* values of 821.481, 855.492, 1163.640, and 1127.216, and doubly charged ions at *m/z* 839.477, 897.509, 919.518, 940.533, 1309.844, 1330.8184, and 1351.832 were observed. These ions could not be annotated by either tandem MS or comparing their accurate masses against external databases. Further identification is needed for these components.

In *E. densa* epidermal cells, the malate abundance distribution was trimodal (see Figure 5A). This can be the basis of *post hoc* identification of three distinct subpopulations containing low, medium, and high malate concentrations. For comparison, we determined the malate abundance distribution for cells in the root nodules of *G. max*, infected by *B. japonicum*. These measurements yielded a bimodal distribution corresponding to post hoc identified subpopulations with low and high levels of malate. Malate has a multitude of functions in plant physiology and metabolism. Not only does it play a functional role in the C_4_ pathway but acts as a pH homeostasis regulator, is an intermediate in the TCA cycle, and involved in plant nutrition (Schulze et al., 2002; Finkemeier and Sweetlove, 2009). These identified subpopulations may be related to different malate levels in quiescent cells (G0), and cells participating in the cell cycle.

Our results illustrate the feasibility of utilizing f-LAESI-MS to probe cellular heterogeneity directly from tissue-embedded single cells. Metabolic noise of primary and secondary metabolites from *E. densa* epidermal cells and infected *G. max* root nodule cells was observed. A rare cell type, specialized excretory idioblasts in *E. densa*, was selectively targeted, i.e., prespecified, for analysis, and compared to other common epidermal cell types. Significant differences were found between their metabolite compositions that otherwise would have been masked by the overall population average. Additionally, based on single cell analysis, differences in the metabolite makeup of epidermal cells in two waterweed species, *E. densa* and *E. canadensis*, were identified based on their unique metabolite profiles and the metabolic noise observed.

For transcripts and proteins, the measured noise, $\eta_{m}^{2}$, consists of technical, $\eta_{t}^{2}$, intrinsic, $\eta_{\mathrm{int}}^{2}$, and extrinsic, $\eta_{\mathrm{ext}}^{2}$, components (Swain et al., 2002):

(3) $$\eta_{\text{m}}^{2}=\eta_{\text{int}}^{2}+\eta_{\text{ext}}^{2}+\eta_{\text{t}}^{2}.$$

The intrinsic noise is associated with the transcription and translation processes, and it becomes dominant for low copy number proteins (below 10 copies/cell) as it follows a trend close to the inverse of the copy number (Taniguchi et al., 2010). At higher copy numbers (above 10 copies/cell), the extrinsic noise presents a floor that no longer depends on protein copy numbers. Extrinsic noise originates from all other sources of fluctuations, e.g., the number of ribosomes in the cell, and the stage in the cell cycle.

Copy numbers of transcripts, proteins, and metabolites in a typical 1 fL *E. coli* cell are in the range of 1–100, 1–300,000, and 100–10^8^, respectively, whereas for a 20 pL *A. thaliana* epidermal cell, they are 1–10^8^, ≤3 × 10^8^, and 10^9^–7 × 10^12^, respectively (Zhang and Vertes, 2018). Therefore, intrinsic stochastic effects can be significant in microorganisms, and perhaps in the genetic regulation of plants, but are unlikely to play a role in plant metabolic processes. In particular, as metabolic enzymes typically have copy numbers higher than 10 copies/cell, the intrinsic noise can be neglected. In our experiments, for metabolites the technical noise, $\eta_{t}^{2}$ ≈ 0.02, is also negligible, and the measured noise is mainly the consequence of the extrinsic noise, $\eta_{m}^{2}$ = $\eta_{\mathrm{ext}}^{2}$. However, it is unclear how the noise from extrinsic sources propagates through the complex system of metabolic pathways.

Table 1 compares the measured metabolic noise values for primary and secondary metabolites in *E. densa* epidermal cells and *G. max* infected root nodule cells. Whereas some primary metabolites showed lower noise in the latter, both cell types exhibited higher noise for secondary metabolites. This observation can be explained by the generally lower copy numbers for secondary metabolites and tighter regulation of the vital primary metabolites.

Based on their metabolite compositions determined by f-LAESI-MS, we were able to find distinguishing features for prespecified populations of epidermal cells from two closely related waterweed species, *E. densa* and *E. canadensis*. A distinguishing feature (biomarker), only present in the *E. canadensis* epidermal cells and not in *E. densa*, was luteolin diglucuronide and/or its isomers. Indeed, flavonoid profiling of bulk *E. canadensis* tissue had revealed three species-specific flavone-diglucuronide, apigenin-7-*O*-diglucuronide, luteolin-7-*O*-diglucuronide, and chrysoeriol-7-*O*-diglucuronide (Mues, 1983). Two sample *t*-test indicated that ascorbate was significantly more abundant (*p* = 0.0002) in the cells from *E. canadensis* (see lower panel of Figure 6C). Abundances of other metabolites, e.g., glutamate and PG(16:0/18:3), were found not to be significantly different in the two cell types.

An important limitation of the current method is its low throughput. Compared to single cell transcriptomics performed on 100,000 cells, and single cell proteomics, carried out on thousands of cells, the ∼100 cell numbers in achieved by the presented method are significantly lower resulting in lower statistical power. Ongoing work aims to increase throughput in cell targeting and analysis to improve statistical power. For the rapid spatial mapping of metabolite abundances within a tissue, automation of cell sampling by image processing software is underway. Although our study utilized exclusively plant cells to demonstrate the utility of the methods, it is clear that f-LAESI-MS should be widely applicable for the analysis of a variety of cell types (e.g., plant, animal, insect, etc.).

## Author Contributions

AV and SS conceived the study, and SS and RK conducted the experiments. BA grew the G. max plants. SS and AV performed the data analysis and SS and AV wrote the manuscript with input from CA, LP-T, and GS.

## Conflict of Interest Statement

The authors declare that the research was conducted in the absence of any commercial or financial relationships that could be construed as a potential conflict of interest.

## Footnotes

1 https://www.ebi.ac.uk/metabolights/index

2 http://plantcyc.org

3 https://metlin.scripps.edu

4 https://metabolomics.cc.hawaii.edu/software/MetImp/
