# Supplementary material for: Metabolic Noise and Distinct Subpopulations Observed by Single Cell LAESI Mass Spectrometry of Plant Cells in situ
Source: Front Plant Sci. 2018 Nov 15;9:1646. doi: 10.3389/fpls.2018.01646 (PMC6250120; doi:10.3389/fpls.2018.01646)
Supplement: Supplementary file 1 [file Image_1.pdf]

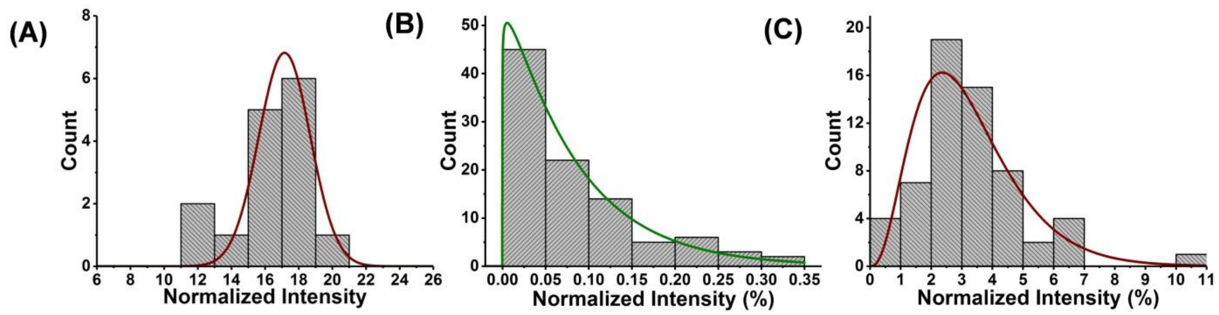

Figure S1. (A) Technical variability for f-LAESI-MS based on sampling 1 μL droplets (n = 15) of 500 μM glutamate standard solution. Results are consistent with a normal distribution and a COV of 14.9%. Single cell results are a convolution of technical and biological variability. For example, sampling **(B)** *E. densa* epidermal cells (n = 97) and **(C)** *G. max* infected root nodule cells (n = 60) revealed gamma distributions with COV values of 97.3% and 50.7%, respectively.
