# Supplementary material for: Metabolic Noise and Distinct Subpopulations Observed by Single Cell LAESI Mass Spectrometry of Plant Cells in situ
Source: Front Plant Sci. 2018 Nov 15;9:1646. doi: 10.3389/fpls.2018.01646 (PMC6250120; doi:10.3389/fpls.2018.01646)
Supplement: Supplementary file 2 [file Table_1.docx]

**Table S1.** Descriptive statistics, goodness of fit, and model parameters for f-LAESI-MS analysis of metabolite abundance distributions for *E. densa* epidermal cells (n = 97) and droplets of glutamate standard solution.

|  | | **Metabolites** | **glutamate std** | **glutamate** | **ascorbate** | **hexose** | **citrate** | **fumarate** | **oxalate** |
| --- | --- | --- | --- | --- | --- | --- | --- | --- | --- |
| **Descriptive statistics** | | **Mean (µ_m_)** | 16.59 | 0.083 | 0.67 | 8.93 | 1.83 | 7.32 | 0.118 |
|  |  | **SD (σ_m_)** | 2.47 | 0.080 | 0.59 | 6.0 | 1.29 | 3.50 | 0.084 |
|  |  | **Non-zero values (%)** | 100 | 73 | 94 | 98 | 100 | 100 | 84 |
|  |  | **COV (%)** | 14.89 | 97.25 | 88.17 | 66.94 | 70.43 | 47.80 | 71.23 |
| **Goodness-of-fit** | **Normal** | **P-value** | 0.50 | N/A | N/A | 0.10 | N/A | 0.89 | 0.10 |
|  |  | **Decision at 5%** | not reject | reject | reject | not reject | reject | not reject | not reject |
|  | **Lognormal** | **P-value** | 0.30 | 0.46 | 0.16 | 0.11 | 0.57 | 0.10 | 0.63 |
|  |  | **Decision at 5%** | not reject | not reject | not reject | not reject | not reject | not reject | not reject |
|  | **Gamma** | **P-value** | 0.39 | 0.51 | 0.70 | 0.07 | 0.47 | 0.82 | 0.86 |
|  |  | **Decision at 5%** | not reject | not reject | not reject | not reject | not reject | not reject | not reject |
| **Normal distribution** | | **Mean (µ)** | 16.59 | N/A | N/A | 8.93 | N/A | 7.32 | 0.118 |
|  |  | **Scale (σ)** | 2.47 | N/A | N/A | 5.98 | N/A | 3.50 | 0.084 |
| **Gamma distribution** | | **Scale (θ = 1/β)** | 0.38 | 0.077 | 0.48 | 5.14 | 0.77 | 2.08 | 0.053 |
|  |  | **Shape (α)** | 42.71 | 1.06 | 1.39 | 1.74 | 2.36 | 3.52 | 2.21 |
