# Supplementary material for: Metabolic Noise and Distinct Subpopulations Observed by Single Cell LAESI Mass Spectrometry of Plant Cells in situ
Source: Front Plant Sci. 2018 Nov 15;9:1646. doi: 10.3389/fpls.2018.01646 (PMC6250120; doi:10.3389/fpls.2018.01646)
Supplement: Supplementary file 3 [file Table_2.docx]

**Table S2.** Descriptive statistics for f-LAESI-MS analysis of metabolite abundances for *E. densa* idioblast cells (n=6).

| **Metabolites** | **malate** | **asparagine** | **citrate** | **hexose** | **PG (16:0/18:3)** | **PG (16:0/18:2)** | **azukisaponin I** | **medicoside I** |
| --- | --- | --- | --- | --- | --- | --- | --- | --- |
| **Mean (µ_m_)** | 0.93 | 0.0085 | 0.16 | 0.51 | 0.39 | 1.28 | 37.7 | 0.22 |
| **SD (σ_m_)** | 2.13 | 0.01 | 0.20 | 0.78 | 0.51 | 1.80 | 2.59 | 0.19 |
| **COV (%)** | 229.0 | 117.6 | 125.8 | 152.9 | 130.7 | 140.6 | 6.87 | 84.7 |
