# Supplementary material for: Metabolic Noise and Distinct Subpopulations Observed by Single Cell LAESI Mass Spectrometry of Plant Cells in situ
Source: Front Plant Sci. 2018 Nov 15;9:1646. doi: 10.3389/fpls.2018.01646 (PMC6250120; doi:10.3389/fpls.2018.01646)
Supplement: Supplementary file 4 [file Table_3.docx]

**Table S3.** Descriptive statistics, goodness of fit, and model parameters for f-LAESI-MS analysis of metabolite abundance distributions for infected *G. max* root nodule cells (n = 60).

|  | | **Metabolite** | **glutamate** | **ascorbate** | **hexose** | **citrate** | **fumarate** | **oxalate** |
| --- | --- | --- | --- | --- | --- | --- | --- | --- |
| **Descriptive statistics** | | **Mean (µ)** | 3.22 | 0.90 | 8.61 | 0.26 | 1.32 | 0.35 |
|  |  | **SD (σ_m_)** | 1.63 | 0.47 | 2.60 | 0.11 | 0.72 | 0.22 |
|  |  | **Non- zero values (%)** | 100 | 98 | 100 | 85 | 85 | 85 |
|  |  | **COV (%)** | 50.65 | 51.59 | 30.15 | 41.36 | 54.63 | 63.79 |
| **Goodness-of-fit** | **Normal** | **P-value** | 0.25 | 0.44 | 0.28 | 0.62 | 0.79 | 0.43 |
|  |  | **Decision at 5%** | not reject | not reject | not reject | not reject | not reject | not reject |
|  | **Lognormal** | **P-value** | 0.11 | 0.64 | 0.74 | 0.08 | 0.51 | 1.00 |
|  |  | **Decision at 5%** | not reject | not reject | not reject | not reject | not reject | not reject |
|  | **Gamma** | **P-value** | 0.39 | 0.81 | 0.65 | 0.06 | 0.56 | 1.00 |
|  |  | **Decision at 5%** | not reject | not reject | not reject | not reject | not reject | not reject |
| **Normal distribution** | | **Mean (μ)** | 3.23 | 0.90 | 8.61 | 0.26 | 1.32 | 0.35 |
|  |  | **Scale (σ)** | 1.63 | 0.47 | 2.60 | 0.11 | 0.72 | 0.22 |
| **Gamma distribution** | | **Scale (θ = 1/β)** | 0.87 | 0.22 | 0.71 | 0.045 | 0.46 | 0.15 |
|  |  | **Shape (α)** | 3.72 | 4.10 | 12.11 | 5.70 | 2.87 | 2.29 |
